# Chemical, Antioxidant and Biological Studies of *Brassica incana* subsp. *raimondoi* (Brassicaceae) Leaf Extract

**DOI:** 10.3390/molecules28031254

**Published:** 2023-01-27

**Authors:** Giuseppe Antonio Malfa, Francesco Pappalardo, Natalizia Miceli, Maria Fernanda Taviano, Simone Ronsisvalle, Barbara Tomasello, Simone Bianchi, Federica Davì, Vivienne Spadaro, Rosaria Acquaviva

**Affiliations:** 1 Department of Drug and Health Science, University of Catania, Viale A. Doria 6, 95125 Catania, Italy; 2 Research Centre on Nutraceuticals and Health Products (CERNUT), University of Catania, Viale A. Doria 6, 95125 Catania, Italy; 3 PLANTA/Autonomous Center for Research, Documentation and Training, Via Serraglio Vecchio 28, 90123 Palermo, Italy; 4 Department of Chemical, Biological, Pharmaceutical and Environmental Sciences, University of Messina, Viale Ferdinando Stagno d’Alcontres 31, 98166 Messina, Italy; 5 Foundation “Prof. Antonio Imbesi”, University of Messina, Piazza Pugliatti 1, 98122 Messina, Italy; 6 Department STEBICEF/Section of Botany, Anthropology and Zoology, University of Palermo, Via Archirafi 38, 90123 Palermo, Italy

**Keywords:** cabbages, polyphenols, carotenoids, oxidative stress, H_2_O_2_, ROS, HPLC/DAD, UPLC-MS/MS, nutraceuticals, botanicals, *Artemia salina* Leach

## Abstract

*Brassica incana* subsp. *raimondoi* is an endemic taxon present in a restricted area located on steep limestone cliffs at an altitude of about 500 m a.s.l. in eastern Sicily. In this research, for the first time, studies on the phytochemical profile, the antioxidant properties in cell-free and cell-based systems, the cytotoxicity on normal and cancer cells by 3-(4,5-Dimethylthiazol-2-yl)-2,5-Diphenyltetrazolium Bromide (MTT) assay, and on *Artemia salina* Leach, were performed. The total phenolic, flavonoid, and condensed tannin contents of the leaf hydroalcoholic extract were spectrophotometrically determined. Ultra-performance liquid chromatography—tandem mass spectrometer (UPLC-MS/MS) analysis highlighted the presence of several phenolic acids, flavonoids, and carotenoids, while High-Performance Liquid Chromatography with Diode-Array Detection (HPLC-DAD) identified various kaempferol and isorhamnetin derivatives. The extract exhibited different antioxidant properties according to the five in vitro methods used. Cytotoxicity by MTT assay evidenced no impact on normal human fibroblasts (HFF-1) and prostate cancer cells (DU145), and cytotoxicity accompanied by necrotic cell death for colon cancer cells (CaCo-2) and hepatoma cells (HepG2), starting from 100 μg/mL and 500 μg/mL, respectively. No cytotoxic effects were detected by the *A. salina* lethality bioassay. In the H_2_O_2_-induced oxidative stress cell model, the extract counteracted cellular reactive oxygen species (ROS) production and preserved non-protein thiol groups (RSH) affected by H_2_O_2_ exposure in HepG2 cells. Results suggest the potential of *B. incana* subsp. *raimondoi* as a source of bioactive molecules.

## 1. Introduction

**Figure 1:**
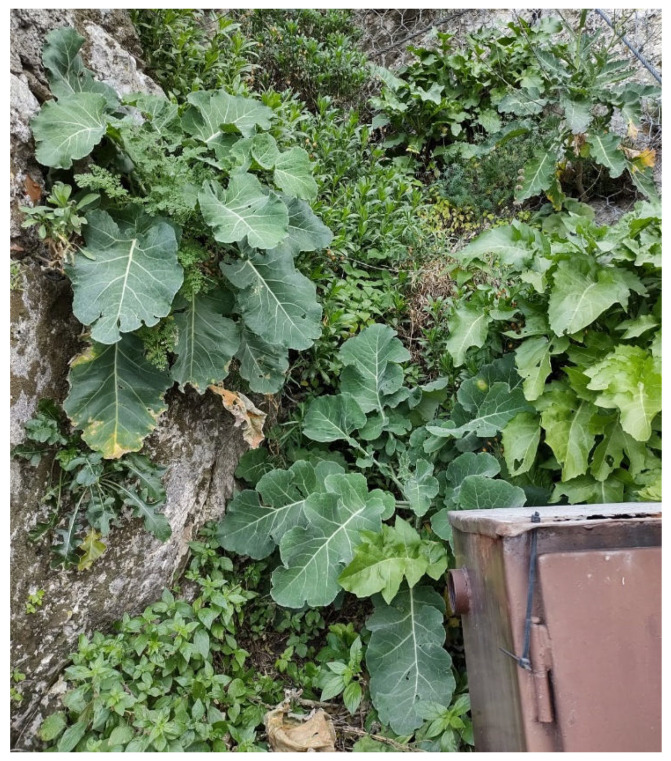
*Brassica incana* subsp. *raimondoi* (Sciandr., C. Brullo, Brullo, Giusso, Miniss. and Salmeri) Raimondo and Spadaro in the respective *locus classicus* (Castelmola, Messina, Italy).

With the term wild cabbages, we mean all those species that grow spontaneously in nature belonging to the *Brassica* genus, whose best-known relatives are domestic cabbages, namely broccoli, cauliflower, kohlrabi, etc., which are simply different cultivated varieties of the same species, known by the botanical name of *Brassica oleracea* L. [1,2]. These plants represent one of the first vegetable food sources of the ancient Mediterranean populations, millennia before foods such as potatoes and tomatoes arrived from the new world [3]. Several species of wild cabbage that grow in the Mediterranean area, particularly Sicily, can be considered the cradle of the species belonging to this genus. Of the total 20 taxa included in the *Brassica* sect. *Brassica* (Brassicaceae) at the specific and intraspecific levels, 13 constitute the *B. oleracea* group or cytodeme, featured by the same diploid genome 2n = 18 (x = 9); among them, 10 are strictly endemic to Sicily [4]. These taxa have been studied and used in crossing programs to select new, improved cultivated forms of cabbages [5,6]. Nowadays, vegetable food such as broccoli or brussels sprouts has a reputation as a superfood for the presence of phytochemicals with beneficial properties [7]. In fact, several studies reported the antioxidant, anticarcinogenic, anti-inflammatory and cardiovascular protective activities of *Brassica* vegetables [8,9]. Wild species belonging to this genus, capable of thriving in nature without human presence, can represent a sustainable and alternative agronomic resource with a strong attraction for the market of health products due to their higher content of bioactive secondary metabolites [4,10]. In general, several plants from this family, thanks to their acknowledged beneficial activities, have found wide use in the production of nutraceuticals and cosmeceuticals [11,12,13]. *Brassica incana* subsp. *raimondoi* (Sciandr., C. Brullo, Brullo, Giusso, Miniss. and Salmeri) Raimondo and Spadaro was described for the first time at specific rank (*B. raimondoi*) by Sciandrello et al. in 2013 [14], and as for other taxa of *Brassica* sect. *Brassica,* it presents a diploid genome 2n = 18. This taxon is present in a restricted area located on steep limestone cliffs at an altitude of about 500 m a.s.l. on the north/east side of the little town of Castelmola (Messina, Italy) (Figure 1).

It is an about 120 cm tall, stout, often branched suffruticose plant, easily identifiable by the firm, light thickened, green-colored lush leaves or by the white color of the large flowers during the flowering season from March to May, while the fruits arise from June to July. *Brassica raimondoi* was reported as a Critically Endangered species according to the criteria of the IUCN Red list due to the small population (<100) and area of distribution [14]. The present experimental study is part of a larger project for the valorization and conservation through the chemical and biological characterization of the active secondary metabolites of nutraceutical interest present in autochthonous species belonging to the genus *Brassica* sect. *Brassica* endemic to Sicily. In particular, for the first time, this study investigated the phytochemical profile limited to polyphenols and carotenoid compounds of an 80% methanolic extract of *B. raimondoi* leaves. In addition, the in vitro cell-free antioxidant characterization, cytotoxicity on normal and cancer cell lines, as well as against *Artemia salina* nauplii, and the potential antioxidant activities of the extract in a cellular system of human hepatocytes were explored.

## 2. Results

### 2.1. Phytochemical Analysis

#### 2.1.1. Determination of Total Polyphenols, Flavonoids, and Condensed Tannins

The polyphenol and flavonoid content of *B. raimondoi* methanolic extract, determined by spectrophotometric methods, was equal to 38.12 ± 0.50 mg gallic acid equivalent (GAE)/g and 8.45 ± 0.60 mg quercetin equivalent (QE)/g extract, respectively. The condensed tannin content was equal to 4.70 ± 0.07 mg of catechin equivalent (CE)/g extract (Table 1).

#### 2.1.2. Ultra-Performance Liquid Chromatography-Mass Spectrometry Analysis

UPLC-MS/MS analysis was performed on *B. raimondoi* leaf extract to characterize its secondary metabolites profile. Specifically, we searched for hydroxycinnamic acids, flavonoids, and carotenoids (Table 2). The presence of flavonoid compounds and hydroxycinnamic acid derivatives was detected by the presence of the molecular ion and its fragments. However, xanthine compounds and carotenoids were below the limit of detection (LOD) and therefore were not detected.

#### 2.1.3. Polyphenolic Profile Characterization by HPLC/DAD Analysis

**Figure 2:**
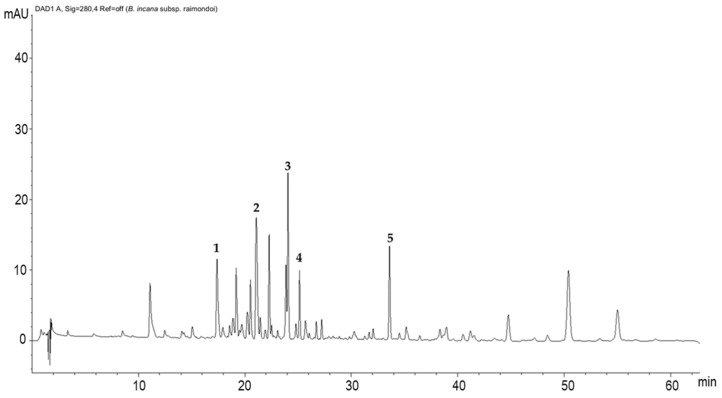
HPLC-DAD polyphenols fingerprint of *Brassica raimondoi* leaf extract (mAU—milli-absorbance unit). Column: Ascentis Express C18, 15 cm × 4.6 mm, 2.7 µm d.p. The numbers indicating peaks refer to the identified compounds reported in Table 3: kaempferol-3-*O*-diglucoside-7-*O*-glucoside (1); kaempferol-3-hdroxyferuloylsophoroside-7-glucoside (2); quercetin-3-feruloyl-diglucoside-7-glucoside (3); isorhamnetin-3-glucoside-7-glucoside (4); kaempferol-rutinoside (5).

The chromatogram recorded at 280 nm (Figure 2) showed ten predominant constituents, accompanied with several minor peaks, and five compounds were identified. Peak 1 was identified as kaempferol-3-*O*-diglucoside-7-*O*-glucoside (17.4 min.), peak 2 was identified as kaempferol-3-hdroxyferuloylsophoroside-7-glucoside (21.08 min.), peak 3 was identified as quercetin-3-feruloyl-diglucoside-7-glucoside (24.06 min.), peak 4 was identified as isorhamnetin-3-glucoside-7-glucoside (25.14 min.), and peak 5 was identified as kaempferol-rutinoside (33.59 min.) (Table 3). Otherwise, all the other peaks not identified are mostly attributable to different polyphenol derivatives.

### 2.2. In Vitro Cell-Free Antioxidant Properties

#### DPPH (2,2-diphenyl-1-picrylhydrazyl), Sod-like Activity, β-Carotene Bleaching, Reducing Power and Chelating Activity Assays

The antioxidant properties of the leaves of *B. raimondoi* methanolic extract were assessed using different tests to determine the possible antioxidant mechanisms of action of the phytocomplex. Table 4 shows the results for the 2,2-diphenyl-1-picrylhydrazyl (DPPH) assay used to test the radical-scavenging ability (IC_50_ 1.33 ± 0.02 mg/mL, IC_50_—half maximal inhibitory concentration); the superoxide dismutase-like activity (SOD-like activity) assay to test the scavenger effect with a method that excludes Fenton-type reactions and the xanthine/xanthine oxidase system (IC_50_ 81.78 ± 2.3 μg/mL) β-carotene bleaching test to determine the antioxidant activity against lipid peroxidation (IC_50_ 28.82 ± 1.73 and 39.48 ± 2.11 μg/mL); and the reducing power assay to evaluate the ability of the extract to donate an electron (13.22 ± 0.60 ascorbic acid equivalent (ASE)/mL). In the ferrous ion (Fe^2+^) chelating activity assay, the extract did not exhibit any chelating properties.

### 2.3. Toxicity

#### 2.3.1. Cytotoxicity on Normal and Cancer Cells by MTT Assay

**Figure 3:**
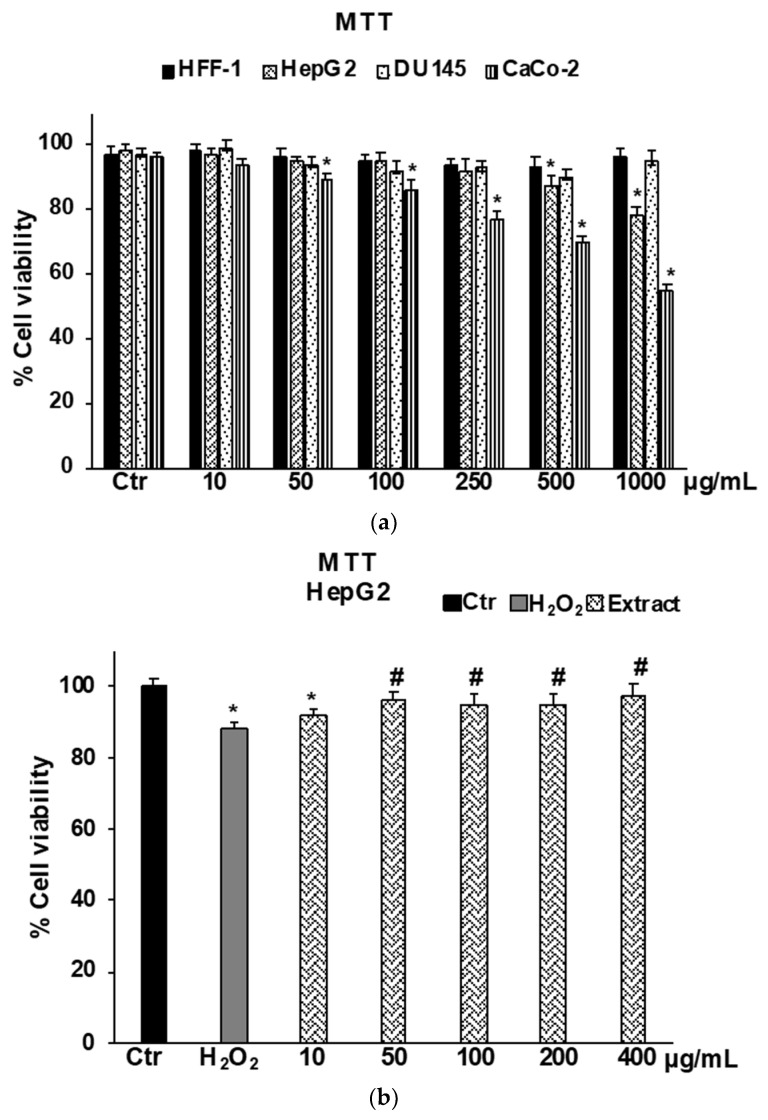
Cell viability in HFF-1, HepG2, DU145, and CaCo-2 cells untreated (Ctr), and treated with *Brassica raimondoi* extract for 24 h (**a**). Cell viability in HepG2 cells untreated (Ctr), treated for 2 h with H_2_O_2_ 200 μM (H_2_O_2_), and pre-treated with the extract (10–50–100–200–400 μg/mL) followed by H_2_O_2_ treatment (200 μM) for 2 h (**b**). Values are the mean ± S.D. of five experiments in triplicate. Confidence intervals calculated by two-way ANOVA test: * Significant vs. untreated control cells: *p* < 0.001; # Significant vs. H_2_O_2_ treated cells: *p* < 0.001.

MTT assays performed in healthy normal human foreskin fibroblast cells (HFF-1) highlighted that *B raimondoi* extract at all concentrations tested (10–1000 μg/mL) did not affect cell viability (Figure 3A). Results on human liver carcinoma cells HepG2, a cell line largely used for drug development and toxicity testing, showed that the extract affects cell viability starting from the concentration of 500 μg/mL. At the lowest concentrations the extract did not affect cell viability, but it was able to exert a protective effect on HepG2 cells exposed to H_2_O_2_ used as an in vitro model of oxidative stress (Figure 3B). The concentrations ranging from 10 to 400 μg/mL in this cell line were used in further investigations on the antioxidant effect in a cell system. The exposure of the cancer cell lines to *B. raimondoi* extract for 24 h produced a cytotoxic effect only on CaCo-2 cells (Figure 3A) (IC_50_ doxorubicin, 21 ± 0.3 μM); contrarily, no effect was detected on DU-145 at all tested concentrations (10–1000 μg/mL) (Figure 3A).

#### 2.3.2. LDH Release

**Figure 4:**
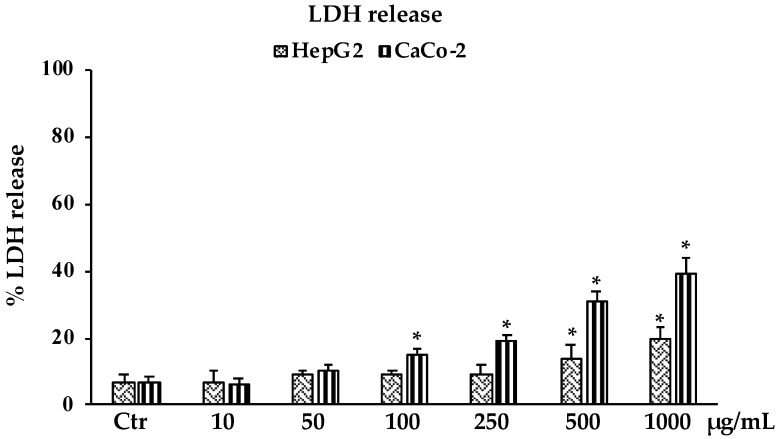
LDH release in untreated HepG2 and CaCo-2 cells (Ctr) and treated for 24 h with the extract (10–1000 μg/mL). Values are the mean ± S.D. of five experiments in triplicate. Confidence intervals calculated by two-way ANOVA test: * Significant vs. untreated control cells: *p* < 0.001.

Consistent with the results observed in the MTT assay, in Figure 4 is reported the percentage of lactate dehydrogenase (LDH) release in HepG2 and CaCo-2 cells treated with different concentrations of *B. raimondoi* extract. Results clearly showed that the extract affected the viability of both cell lines by inducing necrotic cell death. The highest values, of about 20% and 40%, were reached at the highest tested concentration of 1000 μg/mL for HepG2 and CaCo-2 cells, respectively.

#### 2.3.3. Toxicity Assessment by Artemia salina Leach Lethality Bioassay

The results of the *A. salina* lethality bioassay carried out for the extract of *B. raimondoi* showed the absence of toxicity against brine shrimp larvae. Indeed, after 24 h of exposure to the extract, all the larvae were alive for all tested concentrations (10, 100, 500 and 1000 µg/mL); therefore, the median lethal concentration is higher than 1000 µg/mL, which indicates the potential safety of the extract according to the Clarkson’s toxicity scale [15].

### 2.4. Determination of Reactive Oxygen Species (ROS) and Non-Protein Thiol groups (RSH) in Oxidative Stress H_2_O_2_-Induced HepG2 Cells

**Figure 5:**
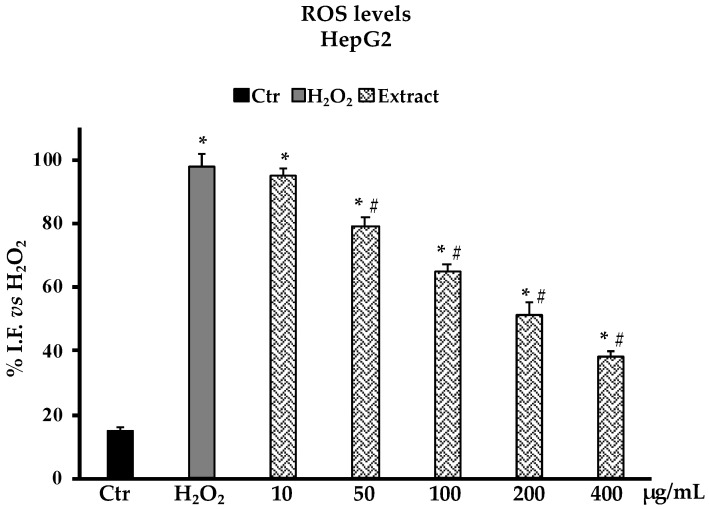
ROS production in HepG2 untreated cells (Ctr), treated for 2 h with H_2_O_2_ 200 μM (H_2_O_2_), and pre-treated with *Brassica raimondoi* extract (10–50–100–200–400 μg/mL) and followed by H_2_O_2_ treatment 200 μM 2 h. Results are expressed as percentage of fluorescence intensity (I.F.)/mg protein. Values are the mean ± S.D. of five experiments in triplicate. Confidence intervals calculated by two-way ANOVA test: ^*^ Significant vs. untreated control cells: *p* < 0.001; # Significant vs. H_2_O_2_ treated cells: *p* < 0.001. I.F. vs. H_2_O_2_.: Intensity of Fluorescence vs. H_2_O_2_ treated cells.

**Figure 6:**
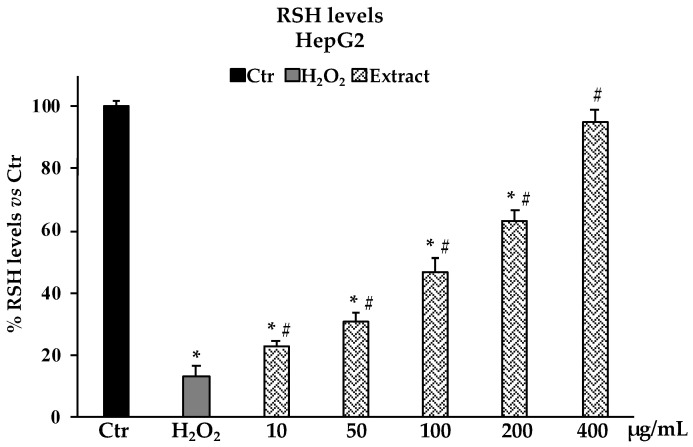
RSH (non-protein thiol groups) levels in HepG2 untreated cells (Ctr), treated for 2 h with H_2_O_2_ 200 μM (H_2_O_2_), and pre-treated with *Brassica raimondoi* extract (10–50–100–200–400 μg/mL) and followed by H_2_O_2_ treatment 200 μM 2 h. Values are the mean ± S.D. of five experiments in triplicate. Confidence intervals calculated by two-way ANOVA test: ^*^ Significant vs. untreated control cells: *p* < 0.001; # Significant vs. H_2_O_2_ treated cells: *p* < 0.001. % RSH levels vs Ctr.: percentage of non-protein thiol groups vs. untreated control cells.

The determination of ROS production in HepG2 cells as a model system for oxidative H_2_O_2_-induced stress revealed that pretreatments with the *B. raimondoi* extract at the different concentrations tested (10–400 µg/mL), starting from 50 µg/mL, were able to reduce the number of free radicals in cells in a dose-dependent manner. At 200 µg/mL, the presence of ROS is reduced by about 50% with respect to non-pre-treated cells (Figure 5). In accordance with the above results, the investigation on non-protein thiol groups content after H_2_O_2_ exposure in HepG2 cells pretreated with different concentrations of *B. raimondoi* (10–400 µg/mL) showed a significative protective effect on thiol groups most represented by glutathione (GSH) (Figure 6). At 400 µg/mL, the extract was able to fully restore the RSH content at level comparable to untreated control cells (Figure 6).

## 3. Discussion

Sicily is an extraordinary country rich in plant endemism, representing about 10% of the total flora of the region [16]. This is mainly due to the isolation of the territories and the variety of environments and substrates distinctive to the island [17]. The study of these unique species is a substantial aspect of botanic research for preserving biodiversity, ecology, and genetic resources. Even the analysis of the secondary metabolites profile and the evaluation of their biological activities defines a taxon, especially when it is edible or with medicinal properties [18]. Many of these species are indeed promising candidates as alternative crops if domesticated, representing a sustainable resource for the local economies and the market of botanicals and nutraceuticals [4]. In this context, *B. raimondoi* represents an interesting objective of study given the growing interest in *Brassica* vegetables and derived products, now commonly considered a superfood due to their nutritional value and beneficial effects on humans. The health benefits are correlated as well as nutrients, vitamins, and minerals, also to several bioactive molecules such as carotenoids, polyphenols, and glucosinolates, which constitute the main components of the phytocomplex present in *Brassica* species. Determined for the first time in this taxon, the total polyphenols content obtained by the Folin–Ciocalteau method revealed an amount of 38.11 ± 0.50 mg GAE/g, resulting the highest among other extracts of wild taxa of *Brassica* and cultivated varieties of *B. oleracea* L. [9], even if it is comparable to the result reported by Miceli et al. for a leaf hydroalcoholic extract (MeOH 70%) of *Brassica incana* Ten. [19]. In another study, different samples of *B. incana* showed the highest phenolic content with respect to *B. rupestris* and two different Sicilian endemisms, respectively—*B. macrocarpa* and *B. villosa* [20]. Further phytochemical analysis revealed the presence of numerous polyphenol compounds, mainly represented by simple phenolic acids (caffeic, ferulic, sinapic, and *p*-coumaric acids) and several flavonoids such as cyanidin, isorhamnetin, kaempferol, luteolin and quercetin (Table 2); the total flavonoid content was determined spectrophotometrically in 8.45 ± 0.60 (mg QE/g extract) (Table 1). Among the listed compounds, some derivatives were analyzed by HPLC-DAD and identified as kaempferol-3-*O*-diglucoside-7-*O*-glucoside, kaempferol-3-hdroxyferuloylsophoroside-7-glucoside, quercetin-3-feruloyl-diglucoside-7-glucoside, isorhamnetin-3-glucoside-7-glucoside, and kaempferol-rutinoside (Table 3). All of them are largely present in the phytochemical profile of several wild species of *Brassica* and cultivated varieties of *B. oleracea* [19,20,21]. These molecules possess several biological effects and are associated with decreased risk of developing various diseases [22,23]. It is well documented that their antioxidant properties and radical scavenging capacity reduce ROS generation, preventing the oxidative damage of biological macromolecules in cells, thus exerting anti-inflammatory, cardioprotective, antiaging, and anticancer activities [24]. In addition, the qualitative phytochemical analysis highlighted the presence of numerous carotenoids in the extract of *B. raimondoi*, specifically α-carotene, β-carotene, cryptoxanthin, neoxanthin, violaxanthin, and zeaxanthin (Table 2). These isoprenoid derivatives are object of phytochemical studies and target of breeding programs among *Brassica* species due to their great medicinal value [25]. The antioxidant activity of plant extracts and their biological activities are often explained by their phytochemical profile. The antioxidant tests on *B. raimondoi* leaf extract highlighted primary antioxidant ability (DPPH, IC_50_ 1.33 ± 0.02 mg/mL; SOD-like, IC_50_ 66.66 ± 1.1 µg/mL) (Table 4), which was found to be related mainly to the free radical scavenging properties of the active components of the phytocomplex. The instrumental analysis of the extract revealed the presence of compounds belonging to different chemical classes, i.e., phenolics and carotenoids. Phenolic compounds are well known for their antioxidant properties [26], and, for each of the identified compounds in the extract, the radical scavenging activity in the DPPH test has already been reported in previous works [27,28,29]. In addition, the good antioxidant properties of pure tested carotenoids have been reported by some authors, indicating carotenes as a more efficient antioxidant than xanthophylls [30,31,32]. Despite the promising phytochemical profile of the extract, the SOD-like activity and reducing power test values (Table 4) are lower than those reported recently for *B. drepanensis,* even if from the β-carotene bleaching test. *B. raimondoi* was potentially more active in inhibiting lipid peroxidation than the above-mentioned Sicilian taxon [9]. The brine shrimp lethality bioassay recorded the absence of toxicity for *B. raimondoi* extract. It is a quick and simple alternative animal model highly valued for its application in the toxicity detection of chemicals and natural products; in recent decades, it has been widely used to test the toxicity of plant extracts [15,33]. Several studies evidenced that *Brassica* species possess potent anticancer properties, mainly due to the bioactive molecules present in the phytocomplex, including phenolic acids, flavonoids, and carotenoids, of which intake is strictly correlated to a reduced risk of several chronic diseases, including cancer, due to their multiple mechanisms of action [34,35,36].

The potential cytotoxic effect of *B. raimondoi* leaf extract was tested by MTT assay on healthy human fibroblasts (HFF-1) and in three tumour cell lines, namely HepG2 (liver cancer), DU145 (prostate cancer), and CaCo-2 (colon cancer). No antiproliferative effects resulted in HFF-1 and DU145 cells. Conversely, the treatments for 24 h with the extract exerted cytotoxicity accompanied by necrotic cell death, in HepG2 and CaCo-2 cells. In previous works, similar results have been highlighted for *B. incana* and *B. drepanensis*, hypothesizing the involvement of signalling pathways differently mediated by the phytocomplex [9,19]. Since oxidative stress is a transversal factor in numerous liver disorders, we decided to test the antioxidant properties of the extract in a cell system of oxidative stress induced by H_2_O_2_ on HepG2 cells, a human hepatoma cell line largely used in hepatotoxicity studies [37,38]. *Brassica raimondoi* leaf extract at all the concentrations tested exerted protective effects against H_2_O_2_-induced oxidative stress and counteracted ROS production and preserved non-protein thiol groups (RSH)amount (Figure 5 and Figure 6). The latter is closely related to the content of glutathione (GSH), an endogenous tripeptide with multiple crucial roles in the cell, such as intracellular redox state maintenance, signal transduction, cell proliferation, apoptosis, etc. [39]. Dysregulation in GSH amount, strictly linked with oxidative stress conditions, is involved in aging and in several human diseases, including hepatic disorders [40]. Furthermore, the antioxidant and protective effects of the extract at the tested concentrations were also confirmed by the restoration of cell viability affected by H_2_O_2_ exposure (Figure 3B).

## 4. Materials and Methods

### 4.1. Chemicals

Superoxide dismutase (SOD) and analytic-grade organic solvents were purchased from VWR (Milan, Italy). UHPLC-grade water (18 mW), UHPLC-grade MeOH, and formic acid were purchased from Carlo Erba (Milan, Italy). The reference compounds used in the *B. raimondoi* HPLC analysis were provided by PhytoLab GmbH and Co. (Vestenbergsgreuth, Germany). Unless indicated otherwise, all chemicals were purchased from Sigma-Aldrich (Milan, Italy).

### 4.2. Plant Collection and Extraction Procedure

**Figure 7:**
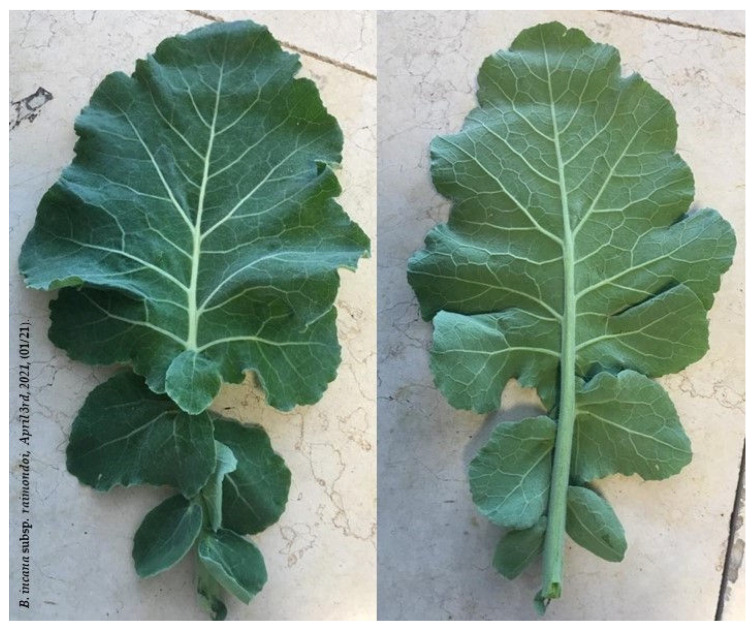
Leaf characteristics and morphology of *Brassica incana* subsp. *raimondoi* (Sciandr., C. Brullo, Brullo, Giusso, Miniss. and Salmeri) used in this research.

The leaves of *Brassica raimondoi* were collected in the locus classicus of the taxon in the area of Castelmola (Messina, Italy). All vegetal material was sampled without affecting plant survival, and leaves at different vegetative growth stages were homogenously picked up from different plants on 3 April 2021. The taxonomic identification was confirmed by the Italian botanist, F.M. Raimondo (PLANTA/Autonomous Center for Research, Documentation and Training, Palermo, Italy). A voucher specimen (01/21) was deposited in the department of Drug and Health Sciences, University of Catania (Figure 7). After harvesting, the plant material was gently washed and wiped, and immediately frozen at −80 °C. Subsequently the vegetal matrix was smashed with liquid nitrogen and subjected to maceration in an 80% MeOH solution in a ratio of 1:10 (*w*/*v*), at 4 °C for 60 min with continuous stirring. The extraction was repeated three times. The resulted solution was then filtered and evaporated under reduced pressure with a rotatory evaporator to obtain a 10-fold concentrated aqueous extract suitable to be lyophilized. The yield of extraction was 5.4%, compared to 100 g of fresh plant material.

### 4.3. Phytochemical Analysis

#### 4.3.1. Spectrophotometric Determination of Total Polyphenols

The quantitative determination of total polyphenols contained in *B. raimondoi* leaf extract was carried out by the Folin–Ciocâlteu colorimetric method [41]. One hundred μL of appropriately diluted sample solution was mixed with 200 μL Folin–Ciocâlteu reagent, 2 mL of distilled water, and 1 mL of 15% sodium carbonate. The samples were incubated for 2 h in the dark at room temperature; then, the absorbances were measured at λ = 765 nm with a spectrophotometer (Shimadzu UV-1601, Milan, Italy). In the blank, the volume of sample solution was replaced with an equal volume of solvent. Gallic acid was used as a standard to construct a calibration curve for quantitatively estimating total polyphenols. The results, obtained from the average of three independent determinations, are reported as mg gallic acid equivalents (GAE)/g extract (dw) ± S.D.

#### 4.3.2. Spectrophotometric Determination of Total Flavonoids

The determination of total flavonoids contained in *B. raimondoi* extract was carried out by using the aluminum chloride colorimetric assay [42]. Five hundred μL of appropriately diluted sample solution was mixed with 1.5 mL MeOH, 100 μL of 10% aluminum chloride, 100 μL of 1 M potassium acetate and 2.8 mL of distilled water. The samples were incubated for 30 min in the dark at room temperature; then, the absorbances were spectrophotometrically measured at λ = 415 nm. In the blank, the volume of 10% aluminum chloride was replaced by the same volume of distilled water. Quercetin was used to construct a calibration curve for the quantitative estimation of total flavonoids and the results, obtained from the average of three independent determinations, are reported as mg quercetin equivalents (QE)/g extract (dw) ± S.D.

#### 4.3.3. Spectrophotometric Determination of Condensed Tannins

The condensed tannin content of *B. raimondoi* extract was assessed by using the vanillin-HCl colorimetric assay [43]. Fifty μL of properly diluted sample solution was mixed 1.5 mL of 4% vanillin in MeOH; then, 750 μL of concentrated hydrochloric acid was added. The samples were incubated for 20 min at room temperature in the dark and the absorbances were spectrophotometrically measured at λ = 500 nm. In the blank, the volume of 4% vanillin was replaced by the same volume of MeOH. (+)-Catechin was used to make the calibration curve for quantitatively determining condensed tannins. The results, obtained from the average of three independent determinations, are reported as mg catechin equivalent (CE)/g extract (dw) ± S.D.

#### 4.3.4. Ultra-Performance Liquid Chromatography-Mass Spectrometry (UPLC-MS/MS)

Ultra-performance liquid chromatography coupled with mass spectrometry (UPLC-MS/MS) (Perkin-Elmer/AB SCIEX API 2000TM, Shelton, CT, USA) was performed to investigate the presence of polyphenolic and carotenoid compounds in *B. raimondoi* extract. The separation was performed using water with 0.1% formic acid: methanol as the mobile phase. The gradient elution was performed as follows: 0–30 min. 50:50 (*v*/*v*); 30–60 min. 70:30 (*v*/*v*); 60–90 min. 50:50 (*v*/*v*). The elution rate was 300 µL/min for 90 min into a C18 column (Phenomenex Kinetex^®^, Torrance, CA, USA, 6 µm C18 100 Å, 100 × 2.1 mm), with a volume of injection of 10 µL. ESI-MS/MS was used in positive and negative polarities. Other settings were: Curtain Gas (CUR) 25.0; IonSpray Voltage (IS) 4500.0; Temperature (TEM) 300 °C; Ion Source Gas1 (GS1) 25.0; Ion Source Gas2 (GS2) 50.0; Declustering Potential (DP) 80.0; Focusing Potential (FP) 400.0 and Entrance Potential (EP) 10.0.

#### 4.3.5. High-Pressure Liquid Chromatography Diode-Array Detection (HPLC-DAD)

The polyphenolic fingerprinting of the extract was defined by HPLC-DAD. The analyses were performed as described in Malfa et al. 2020 [44]. Specifically, 5 µL of dimethylformamide/water (9:1) sample solution (10 mg/mL) was analyzed in duplicate by HPLC-DAD using a Shimadzu LC 20 (Kyoto, Japan), equipped with a diode array detector (DAD) and with a 150 × 4.6 mm i.d., 2.7 µm Ascentis Express C 18 column maintained at 25 °C with a flow of 1 mL/min. Two mobile phases: H_2_O/H_3_PO_4_ (99:1, solvent A) and MeOH/ACN/H_3_PO_4_ (49.5:49.5:1 solvent B), were used with the following gradient: concentration of solvent A of 95% going to 77% (34 min.), maintained at 77% (3 min.), 74% (60 min.), 60% (85 min.), 20% (90 min.) and 0% (92 min.); total time 105 min. The chromatogram was recorded at 330 nm ± 2 nm.

### 4.4. In Vitro Antioxidant and Free Radical Scavenging Activity

#### 4.4.1. 2,2-diphenyl-2-picrylhydrazyl (DPPH) Assay

The free radical scavenging activity of *B. raimondoi* leaf extract was determined by the 2,2-diphenyl-2-picrylhydrazyl (DPPH) assay [45]. Butylated hydroxytoluene (BHT) was utilized as positive control. An aliquot of 500 µL of each sample solution (0.0625–2 mg/mL) was added to 3 mL of DPPH methanol solution (0.1 mM). The reactive solutions were maintained at room temperature in the dark for 20 min., and then absorbances were measured at λ = 517 nm with a spectrophotometer (Shimadzu UV-1601, Milan, Italy). Three independent experiments were carried out and the results are reported as mean radical scavenging activity (%) ± S.D. and mean 50% inhibitory concentration (IC_50_) ± S.D.

#### 4.4.2. SOD-Superoxide Dismutase like Activity

The scavenger activity of the extract on superoxide anion was evaluated spectrophotometrically according to the method previously reported by Genovese et al. [46]. The method involves the in vitro production of the superoxide anion with the consequent oxidation of nicotinamide adenine dinucleotide (NADH), detected by measuring the decrease in its absorbance at 340 nm. The results are expressed as percentage inhibition of NADH oxidation, and SOD (80 mU) was used as the reference compound.

#### 4.4.3. β-Carotene Bleaching Test

The β-carotene bleaching test was performed as previously described [9]. The final solution of linoleic acid, Tween 20, and β-carotene was added to a 96-well microplate containing samples in the concentration range of 2.5–100 µg/mL and further incubated at 45 °C. The absorbance was measured at λ = 470 nm at T = 0 and after 30 and 60 min of incubation. Propyl gallate was used as reference compound.

#### 4.4.4. Reducing Power Assay

The reducing power of *B. raimondoi* extract was evaluated by the Fe^3+^–Fe^2+^ transformation method, according to the method of Oyaizu et al. [47]. Butylated hydroxytoluene (BHT) and ascorbic acid were utilized as standard reference drugs. For the test, 1 mL of each sample solution (0.0625–2 mg/mL) was mixed with 2.5 mL of phosphate buffer (0.2 M, pH 6.6) and 2.5 mL of 1% potassium ferricyanide. The samples were incubated for 20 min at 50 °C and, after rapid cooling, 2.5 mL of 10% trichloroacetic acid was added. The mixture was centrifuged for 10 min at 3000 rpm at a temperature of 4 °C; then, 2.5 mL of the solution taken from the upper layer was mixed with 2.5 mL of distilled water and 500 µL of 0.1% ferric chloride. In the blank, the volume of sample solution was replaced with an equal volume of solvent. Following incubation for 10 min in the dark at room temperature, the absorbances of the solutions were measured at λ = 700 nm. Three independent experiments were carried out and the results are reported as mean absorbance values ± S.D. and ascorbic acid equivalent (ASE)/mL.

#### 4.4.5. Ferrous Ion (Fe^2+^) Chelating Activity

The chelating activity of *B. raimondoi* extract was estimated by the spectrophotometric measurement of the Fe^2+^–ferrozine complex, according to the method previously reported by Kumar and colleagues (2008), with slight modifications [48]. Ethylenediaminetetraacetic acid (EDTA) was utilized as positive control. To an aliquot of 1 mL of sample solution (0.0625–2 mg/mL), 50 µL of 2 mM ferrous chloride and 500 µL of distilled water were added. One hundred µL of 5 mM ferrozine solution was added to initiate the reaction; following incubation for 10 min at room temperature in the dark, the absorbances of the solutions were measured at λ = 562 nm. Three independent experiments were carried out and the results are reported as mean inhibition of the ferrozine–(Fe^2+^) complex formation (%) ± S.D. and IC_50_ ± S.D.

### 4.5. Cell Culture

Human foreskin fibroblasts (HFF-1) were obtained from the American Type Culture Collection (SCRC-1041, ATCC^®^, Rockville, MD, USA). HFF-1 were cultured in Dulbecco’s Modified Eagle’s Medium (DMEM) supplemented with 15% fetal bovine serum (FBS), 4.5 g/L glucose, 100 units/mL penicillin, and 100 μg/mL streptomycin. The human nontumorigenic hepatocytes HepG2 (HB-8065, ATCC^®^, Rockville, MD, USA) were maintained in Minimum Essential Medium (MEM) containing 10% FBS, 100 units/mL penicillin, 100 μg/mL streptomycin, and 2 mm glutamine. Human colon carcinoma cells CaCo-2 (HTB-37, ATCC^®^, Rockville, MD, USA) were cultured in DMEM supplemented with 10% FBS, 1 mmol/L sodium pyruvate, 2 mmol/L L-glutamine, streptomycin (50 μg/mL) and penicillin (50 U/mL). Prostate cancer DU145 cells (HTB-81, ATCC^®^, Rockville, MD, USA) were cultured in RPMI supplemented with 5% FBS, 100 units/mL of penicillin, and 100 μg/mL of streptomycin. All cell lines were cultured in a humidified atmosphere, in 5% CO_2_ at 37 °C, and at sub-confluent conditions they were plated at a constant density to obtain identical experimental conditions in the different tests.

### 4.6. Cytotoxicity Assays

#### 4.6.1. MTT Assay

Cell viability was performed by MTT assay that measures the conversion of tetrazolium salt to yield colored formazan in the presence of metabolic activity [49]. Briefly, cells were plated on a 96 multiwell plate (8 × 10^3^ cells/well) and after 24 h were treated with the different concentrations of *B. raimondoi* extract (10–1000 μg/mL) for 24 h. Differently, in HepG2 as an in vitro model of oxidative stress only non-cytotoxic concentrations (10–400 μg/mL) were tested. The absorbance was measured at λ = 570 nm using a microplate reader (Titertek Multiskan, Flow Laboratories, Helsinki, Finland) and the results are expressed as percentage of cell viability compared to untreated cells (control group).

#### 4.6.2. Lactate Dehydrogenase Release

The LDH activity was measured spectrophotometrically at λ = 340 nm in the culture medium and in the cellular lysates separately by measuring the reduction of β-nicotinamide-adenine dinucleotide (NADH) [50]. Cells were plated on a 6 multiwell plate (4 × 10^4^ cells/well) and after 24 h were treated with the different concentrations of *B. raimondoi* extract (10–1000 μg/mL) for 24 h. The increase of LDH activity in the culture medium shows a relationship with the percentage of dead cells. Results are expressed as the percentage of LDH released.

#### 4.6.3. *Artemia salina* Leach Lethality Bioassay

To predict the toxicity of *B. raimondoi* leaf extract, the brine shrimp (*Artemia salina* Leach) lethality bioassay was performed, following the procedure reported by Meyer and colleagues, with slight modifications [51]. Brine shrimp eggs were hatched in a brine shrimp hatchery dish filled with artificial seawater (32 g/L) under a 60 W lamp, at a temperature of 24–26 °C. At 24 h after hatching, active nauplii free from eggshells were collected from the brighter portion of the hatchery dish and used for the assay. Ten brine shrimp larvae were transferred to plates and incubated in 5 mL of artificial seawater mixed with different amounts of extract (10–1000 µg/mL) at 24–26 °C. After 24 h, the surviving larvae were counted using a magnifying glass, and the median lethal concentration (LC_50_) value was determined. For each sample concentration, three replicates were used. The toxicity level of the extract was assessed according to the Clarkson’s toxicity scale; extracts providing LC_50_ values greater than 1000 μg/mL are considered non-toxic [15].

### 4.7. Antioxidant Activities in Cell

#### 4.7.1. Reactive Oxigen Species Determination

Cells were plated on a 6 multiwell plate (4 × 10^4^ cells/well) and after 24 h were treated with the different concentrations of *B. raimondoi* extract (10–400 μg/mL) for 24 h and followed by H_2_O_2_ treatment 200 μM 2 h. ROS determination was carried out, as previously described [52], by fluorometric evaluation of 2′,7′-dichlorofluorescein (DCF) (excitation, λ = 488 nm; emission, λ = 525 nm) production from the oxidation of 2′,7′-dichlorofluorescein diacetate (DCFH-DA). The fluorescence intensity was normalized with the amount of protein in the sample and evaluated by measuring the absorbance difference at λ = 280 and λ = 260 with the Biotek Sinergy HTX Multi-Mode Microplate Reader (Biotek Instruments, Winooski, VT, USA). Results are expressed as fluorescence intensity/mg protein.

#### 4.7.2. Non-Protein Thiol Groups Determination

Cells were plated on a 6 multiwell plate (4 × 10^4^ cells/well) and after 24 h were treated with the different concentrations of *B. raimondoi* extract (10–400 μg/mL) for 24 h and followed by H_2_O_2_ treatment 200 μM 2 h. RSH groups were measured by evaluating spectrophotometrically at λ = 412 nm the reduction of the chromophore 5,5-ditiobis-2-nitrobenzoic acid (DTNB), as previously described [44]. A calibration curve was obtained using known amounts of GSH and results were expressed as percentage of RSH with respect to the control and measured as nmoles of RSH/mg protein. Protein content was determined using the Sinergy Biotek Sinergy HTX Multi-Mode Microplate Reader (Biotek Instruments, Winooski, VT, USA) by measuring the absorbance difference at λ = 280 and λ = 260.

### 4.8. Statistical Analysis

For the statistical data analysis, one-way and two-way ANOVA methods and Holm methods as post hoc tests were used. In all reported data, the value of * *p* < 0.001 was considered significant. All statistical analyses were carried out with MedCalc version 20.215 (MedCalc Software, Ostend, Vlaanderen, Belgium).

## 5. Conclusions

As said formerly, the research on plant endemisms, including the study of the phytochemical profile and the evaluation of their biological activities, is a significant field of botanic research, especially for those edible or with medicinal properties. These species, if domesticated, represent a sustainable resource for the local economies and the market of botanicals and nutraceuticals. In this study we investigated for the first time the phytochemical profile and some biological activities of a leaf methanolic extract from *B. raimondoi*, punctiform endemism of eastern Sicily (Italy). Phytochemical qualitative studies evidenced the presence of numerous phenolic acids, flavonoids, and carotenoids, all ubiquitous compounds in *Brassica* species. The moderately good antioxidant properties highlighted by in vitro cell-free tests were confirmed further by the protective effects exerted by the extract in a cell system of H_2_O_2_-induced oxidative stress. Although no toxicity against brine shrimp larvae and human fibroblasts was evidenced, indicating the potential safety of the extract, evident cytotoxicity and necrotic cell death were recorded on colon cancer cells and to a lesser degree on hepatocellular carcinoma cells.

Overall, the present findings increased the knowledge of *B. raimondoi*, a little taxon in the Brassicaceae family, which has great potential as a new and safe source of bioactive compounds given the growing interest in *Brassica* vegetables and derived products. They are almost commonly considered superfoods due to their nutritional value and beneficial effects on humans.

## Figures and Tables

**Table 1:** Spectrophotometric quantitative determination of total polyphenols, flavonoids, and condensed tannins in *B. raimondoi* leaf extract.

Total Polyphenols	Total Flavonoids	Condensed Tannins
38.12 ± 0.50	8.45 ± 0.60	4.70 ± 0.07
(mg GAE/g extract) ^1^	(mg QE/g extract) ^1^	(mg CE/g extract) ^1^

^1^ Values, expressed as mg gallic acid (GAE), quercetin (QE) and catechin (CE) equivalents, are the mean ± S.D. of three experiments in triplicate.

**Table 2:** List of polyphenols and carotenoids compounds detected in *Brassica raimondoi* leaf extract by UPLC-MS/MS analysis. Limit of detection (LOD) calculated: 568.2 cps (count per second). Limit of quantitation (LOQ) calculated: 1894 cps (count per second).

Compund	Mol. Weight	Polarity	*m*/*z* (g/mol)	Intensity (cps)
Caffeic Acid	180.16	181.05 (+) 179.04 (−)	179.04	3.5537 × 10^7^
Cryptoxanthin	552.89	553.44 (+) 551.43 (−)	553.44	LOQ *
Cyanidin	287.2442	288.06 (+) 286.05 (−)	286.05	LOQ *
Ferulic Acid	194.184	195.07 (+) 193.05 (−)	193.05	9.9457 × 10^6^
Isorhamnetin	316.2623	317.07 (+) 315.07 (−)	315.07	5.1753 × 10^6^
Kaempferol	286.23	287.06 (+) 285.07 (−)	285.07	5.8504 × 10^6^
Luteolin	286.24	287.06 (+) 285.04 (−)	285.04	5.8504 × 10^6^
Neoxanthin	600.87	601.43 (+) 599.41 (−)	599.41	LOQ *
*p*-coumaric acid	164.158	165.06 (+) 163.04 (−)	163.04	7.7855 × 10^6^
Quercetin	302.24	303.1 (+) 301.04 (−)	301.03	4.5603 × 10^6^
Sinapic Acid	224.21	225.08 (+) 223.06 (−)	223.06	6.5254 × 10^6^
Violaxanthin	600.87	601.43 (+) 599.41 (−)	599.41	LOQ *
Zeaxanthin	568.88	569.44 (+) 567.42 (−)	567.42	LOQ *
α-carotene	536.87	537.45 (+) 535.43 (−)	535.43	LOQ *
β-carotene	536.87	537.45 (+) 535.43 (−)	535.43	LOQ *

* LOQ Limit of Quantification; +: positive polarity, −: negative polarity.

**Table 3:** Polyphenols identified in *Brassica raimondoi* leaf extract by HPLC-DAD.

Peak	Compound	Wavelength (nm)	Ret Time * (min.)
1	kaempferol-3-*O*-diglucoside-7-*O*-glucoside	330	17.4
2	kaempferol-3-hdroxyferuloylsophoroside-7-glucoside	330	21.08
3	quercetin-3-feruloyl-diglucoside-7-glucoside	330	24.06
4	isorhamnetin-3-glucoside-7-glucoside	330	25.14
5	kaempferol-rutinoside	330	33.59

* Retention time.

**Table 4:** Antioxidant activity in cell-free systems.

	DPPH Test IC_50_ (mg/mL)	SOD-like Activity IC_50_ (μg/mL)	β-Carotene Bleaching Test IC_50_ (μg/mL)		Reducing Power ASE/mL
*B. raimondoi*	1.33 ± 0.02	81.78 ± 2.3	28.82 ± 1.73	39.48 ± 2.11	13.22 ± 0.60
Positive control					
BHT	0.07 ± 0.01				0.89 ± 0.06
SOD		40 mU ± 0.85			
Propyl gallate			0.09 ± 0.04	0.09 ± 0.04	

IC_50_: half maximal inhibitory concentration; ASE: ascorbic acid equivalent; BHT: butylated hydroxytoluene; U: enzyme unit; Values are the mean ± S.D. of three experiments in triplicate.

## Data Availability

The data presented in this study are available on request from the authors.

## Sample Availability

Samples of the tested extract are available on request from the authors.
